# Evaluation of subjective visual vertical and horizontal in patients with acoustic neuroma based on virtual reality

**DOI:** 10.3389/fnins.2023.1264585

**Published:** 2023-10-26

**Authors:** Lin Zhang, Shunlin Ouyang, Ling Chen, Hemei Huang, Yongkang Ou, Xiaowu Tang

**Affiliations:** ^1^Department of Otorhinolaryngology, The Third Affiliated Hospital of Guangzhou Medical University, Guangzhou, China; ^2^Department of Otorhinolaryngology, Head and Neck Surgery, Sun Yat-sen Memorial Hospital, Sun Yat-sen University, Guangzhou, China; ^3^Institute of Hearing and Speech-Language Science, Sun Yat-Sen University, Guangzhou, China; ^4^The Seventh Affiliated Hospital, Sun Yat-sen University, Shenzhen, China

**Keywords:** subjective visual vertical, subjective visual horizontal, acoustic neuroma, virtual reality, vestibular function

## Abstract

**Objective:**

To investigate potential differences in absolute deviation values of subjective visual vertical and horizontal between unilateral acoustic neuroma patients and healthy young adults under varying degrees of static head tilt, as well as the impact of proprioception on these values, with the aim of determining the effect of acoustic neuroma on gravity sensory pathway function in patients.

**Methods:**

We recruited 22 patients diagnosed with unilateral acoustic neuroma and 25 healthy young adults and employed virtual reality technology to assess the absolute deviation values of subjective visual vertical (SVV) and subjective visual horizontal (SVH) under eight different static tilted head positions (Head centered (0° tilt), PdP, Head tilt 15°, 30°, 45° to the left and right), then compare and analyze intergroup differences.

**Results:**

In the Head-centered position, both SVV and SVH absolute deviated values were significantly higher in the AN group compared to healthy young adults. The AN group exhibited significantly higher absolute deviation values of SVV compared to the healthy group when tilting their head 30° left and right. Additionally, when tilting their heads to the right at 15° and 45° the AN group showed significant increases in SVH absolute deviated values compared to healthy adults. The SVV and SVH absolute deviation values of LAN and SAN groups did not reach statistical significance. The results of the SVV test for PDP position did not show any significant differences among all groups. However, the SVH test revealed that the absolute deviation values of the LAN group was significantly higher than that of healthy individuals.

**Conclusion:**

Our study shows that the gravity sensing function of patients with unilateral acoustic neuroma is affected to different degrees, however, the degree of gravity sensing function damage of patients has little relationship with tumor size. When acoustic neuroma is larger than 2 cm, the effect of proprioception on patients’ SVH outcome is noteworthy. So, we should pay attention to the postoperative follow-up of patients with acoustic neuroma and the evaluation of vestibular rehabilitation effect. Meanwhile, for patients opting for conservative treatment, it is imperative to monitor the dynamic changes in vestibular function and seize timely opportunities for intervention.

## Introduction

Acoustic neuroma (AN) is a benign tumor commonly found in the internal auditory tract and cerebellar horn region, with an annual incidence of approximately 2.66/100,000 in the Asian population according to literature reports (Koo et al., 2018). The clinical characteristics of AN are dependent on tumor size, location, and involvement of adjacent nerve functions. Common symptoms of acoustic neuroma include hearing loss, tinnitus, vertigo, and balance dysfunction (Humphriss et al., 2006; Huang et al., 2013; Hunter et al., 2016; Lees et al., 2018; Nilsen et al., 2019). However, vestibular symptoms such as vertigo and balance dysfunction are considered significant negative factors that impact the long-term quality of life for patients with acoustic neuroma (Myrseth et al., 2006; Carlson et al., 2015). Due to the relatively slow growth of acoustic neuroma, patients tend to undergo vestibular compensation in their daily lives, and as a result, the clinical manifestations of their balance disorders are often less severe than the actual degree of injury (Stangerup et al., 2010; Huang et al., 2013). Consequently, clinicians frequently overlook the evaluation of vestibular function in patients with AN during clinical visits and follow-up appointments. However, with the continuous improvement of AN diagnosis and treatment in recent years, the focus has shifted from reducing mortality and disability rates to minimizing complications, preserving neurological function, and enhancing patients’ quality of life. Meanwhile, advancements in vestibular function detection technology have enabled the qualitative assessment, localization and diagnosis of vestibular function injuries as well as the evaluation of compensatory effects. This has opened up new possibilities for AN patient in terms of tumor origin identification, neural localization, perioperative function evaluation and postoperative rehabilitation guidance (Borgmann et al., 2011; He et al., 2016; Rahne et al., 2018; Hrubá et al., 2019). As a result, there is an increasing focus on assessing and rehabilitating vestibular function in AN patient.

The vestibular system comprises the semicircular canals and the vestibular sac, with the ampullary crest of the former detecting angular acceleration and the latter (consisting of saccule and utricle) sensing linear acceleration in both horizontal and vertical planes during head movement. This sensory information is crucial for maintaining body balance and spatial orientation, including head tilt and pitch, enabling humans to walk upright and perform various activities. The saccule and utricle, as part of the otoliths in the vestibular system, contribute to the perception of gravitational orientation. The vestibulo-ocular reflex for gravitational perception originates from the utricle. Its signal processing proceeds through the vestibular nerve to the primary vestibular nuclear complex, which is considered an internal model of space and verticality that is updated via bottom-up and top-down processes (Glasauer et al., 2018; Dieterich and Brandt, 2019). The subjective visual vertical (SVV) and subjective visual horizontal (SVH) are useful indices for evaluating spatial disorientation in patients with peripheral and/or central vestibular injury (Ashish et al., 2017). Previous studies have demonstrated the diagnostic efficacy of SVV in assessing patients with diverse vestibular disorders (Dieterich and Brandt, 1993; Byun et al., 2010; Kumar et al., 2017), and it is considered a convenient and expeditious clinical tool for evaluating otolith function and gravity-sensing pathway in both peripheral and central vestibular diseases (Dieterich and Brandt, 1992; Gomez Garcia and Jauregui-Renaud, 2003). The angles of deviation in SVV and SVH may serve as indicators of asymmetry in the bilateral utricle and balance during static head tilt (Bohmer, 1999; Clarke et al., 2003; Schonfeld et al., 2010), thus enabling detection of sensitivity to the static tension balance state of the bilateral utricle (Eghlimi et al., 2012). In the general population, the deviation of subjective visual vertical (SVV) angle typically falls within a range of ±2.5° when an individual is in an upright seated position at rest (Akin et al., 2011; Dakin et al., 2018). Ferreira et al. observed that in cases of unilateral anterior court peripheral disease or neuro-nucleus damage, SVV exhibited a tilt toward the affected side. When the injury site is located in or above the unilateral pontine, there is a deviation of subjective visual vertical toward the healthy side. In the presence of thalamic or cerebellar dental nucleus injury, SVV may deviate toward either the affected or unaffected side (Ferreira et al., 2017). The direction of SVV tilt indicates the site of pathology. All the aforementioned studies were conducted by evaluating the functionality of the gravity-sensing pathway in patients under a single upright head position. In this study, our objective was to investigate potential differences in the absolute deviated values of subjective visual vertical and horizontal between unilateral acoustic neuroma patients with varying degrees of head tilt and healthy adults. In order to evaluate the impact of acoustic neuroma on the function of the gravity receptor pathway in patients, analyze its correlation with tumor size, and examine the role of proprioception in patients with acoustic neuroma. Currently, there is a dearth of research in this field both domestically and internationally. To provide clinicians with a reference for evaluating the balance function, prognosis, and rehabilitation strategy formulation of patients with acoustic neuroma after surgery.

## Materials and methods

### Subjects

A total of 22 patients diagnosed with unilateral acoustic neuroma (AN) were recruited from the Otolaryngology Department at Sun Yat-sen Memorial Hospital, Sun Yat-sen University between January 2020 and June 2022. Additionally, a group of 25 healthy young individuals was included as a reference group for normative data analysis.

Twenty-two consecutive patients were diagnosed with acoustic neuroma (AN) based on preoperative internal auditory canal MRI and postoperative pathology. The group consisted of 9 males and 13 females, nine of the acoustic neuromas were on the left side and 13 were on the right side. Aged between 26 and 69 years old with a mean age of 42.91 ± 12.00 years old. Among them, hearing loss was present in 21 cases (95.45%), tinnitus in 16 cases (72.73%), and dizziness, vertigo or balance impairment in 10 cases (45.45%).

There were 25 healthy young participants, comprising of 9 males and 16 females. Their ages ranged from 19 to 23 years old, with a mean age of 19.78 ± 1.45. All had normal hearing in both ears, no history of otitis media or deafness; No history of dizziness or vertigo; No history of neck disease and exhibited normal range of motion in the neck; Visual acuity was clear after correction without any double vision phenomenon; Normal intelligence was observed along with a clear understanding of test requirements and methods.

Exclusion criteria for all enrolled subjects: (1) Those who suffer from severe balance disorders or nausea and vomiting that may impede their ability to cooperate with the test; (2) Individuals with strabismus, double vision, decreased vision, or an inability to clearly see the target line; (3) Subjects with cognitive impairment who are unable to follow instructions; (4) Participants who have consumed alcoholic drinks or sedative and sleeping drugs within 48 h prior to the test.

The classification of acoustic neuroma was based on the Koos standard: Grade I tumors were limited to the internal auditory canal with a maximum diameter of x ≤ 1 cm; Grade II tumors extended into the cerebellopontine angle without involving the brainstem, and had a maximum diameter of x ≤ 2 cm. Grade III tumors occupied the cerebellopontine angle cistern but did not cause displacement of the brainstem, with a maximum diameter of 2 cm < x ≤ 3 cm. A sizable grade IV neoplasm that induces displacement of the brainstem or nerves. Classes I and II are designated as the small acoustic neuroma (SAN) group, while Classes III and IV are classified as the large acoustic neuroma (LAN) group. Among the patients with acoustic neuroma included in this study, tumors ranged from 10 mm to 41 mm in diameter, with an average of (22.36 ± 8.88)mm. The SAN group comprised 10 cases (45.5%) with a mean age of (49.30 ± 12.03) years, while the LAN group consisted of 12 patients (54.5%) with a mean age of (37.58 ± 8.99) years.

The study was approved by the ethical review board of Sun Yat-sen Memorial Hospital of Sun Yat-sen University (No. SYSKY-2023-393-01). Written informed consent was waived because of this retrospective analysis.

### Data collection

The data in this study were collected at the Vertigo Diagnosis and Rehabilitation Center, Department of Otolaryngology, Sun Yat-sen Memorial Hospital, Sun Yat-sen University. A computer-controlled virtual reality system (Verti SVV, ZT-VNG-I, China) was utilized to measure deviated values of subjective visual vertical and horizontal with reference to fixed parameters set by the instrument. The system comprised a wireless control rod and light-occluding goggles that projected a luminous strip onto the subject’s visual field. When donning the goggles, participants were presented with a yellow luminous strip against a black background, oriented at random non-vertical and non-horizontal angles generated by computerized algorithms. The lines persisted continuously. The VR mask features a 5.1-inch screen with a pixel density of 818 pixels per inch and a resolution of 3,840 × 2,160. The luminous lines displayed on the screen have dimensions of approximately 3.825 × 0.192 inches, the apparent distance from the subject to the SVV/SVH luminous line in the virtual reality world is 3.3 m, and the brightness is adjustable, <200 cd/m^2^. The luminous lines can be randomly tilted to either side at an angle generated by the computer system, ranging from 20° to 90°. The wireless control rod is a cylindrical device measuring 122 mm in length and 26 mm in diameter, which can be conveniently held by the subject using one hand. The rod is equipped with a knob at one end for adjusting the angle of the line, while a separate button in the middle serves as an indicator for confirmation. The wireless control rod features a knob design that ensures a minimum accuracy of 0.2 degrees. The actual operating rotation speed is determined by the rotational speed of the subjects. Prior to commencing the examination, the examiner provided a comprehensive explanation of the subject matter to ensure that each participant comprehends the specific protocol. All AN patients underwent preoperative examination. During the SVV test, participants were instructed to maintain an upright seated position in a comfortable chair while holding a wireless control rod. During the head tilt maneuver, a skilled inspector will help ensure proper alignment of the head angle. They were then required to align the yellow line within their visual field with true verticality and subsequently press the confirm button and repeat the test. After completion of the SVV test, a resting period of 30 min was provided to the subjects prior to conducting the SVH test. Similarly, during the SVH examination, the participants were instructed to align the yellow line within their visual field with true horizontal and press the confirm button. And then repeat the test. Upon the subject’s activation of the wireless lever’s confirm button, the computer records the subject’s head position and calculates SVV and SVH value. Upon completion of one adjustment, the system promptly initiates the subsequent adjustment with a new light source angled randomly.

All participants completed the subjective visual vertical and horizontal tests under eight different degrees head tilt conditions, including seated with neutral head position (0° tilt), standing on a foam cushion with neutral head position (Proprioception deprivation, PdP, 0° tilt), leftward tilts of 15°, 30°, and 45° from neutral, and rightward tilts of 15°, 30°, and 45° from neutral. The tests were conducted in the aforementioned sequence, as our preliminary experiment indicated no significant difference between random and sequential testing, to ensure that no head position test was missed. During the head-tilt sessions, participants were instructed to maintain a static position of the head while tilting at the neck and keeping their trunk as upright as possible. To minimize systematic errors during testing, all participants performed 10 times of SVV and SVH in each head tilt position. The mean value of these 10 times was used as the final result for SVV or SVH. A one-minute rest interval was provided between each position to reduce the impact of vestibular fatigue. When recording test results, the software automatically sets the gravity vertical line and horizontal line as the reference point at 0°. In SVV testing, the upper end of the luminous line slanted to the right is considered positive, while the upper end slanted to the left is deemed negative. In SVH testing, a positive result is indicated by an upward tilt of the left end of the luminous line, while a negative result is indicated by a downward tilt of the left end. In computing the mean deviation of the test results, it is obtained by taking the absolute value. The absolute values indicate the magnitude of deviation. A larger absolute value corresponds to a greater degree of deviation. Note that using the absolute value of the deviation means that the direction of the virtual luminous line setting is not included in the statistical processing.

**Figure 1:**
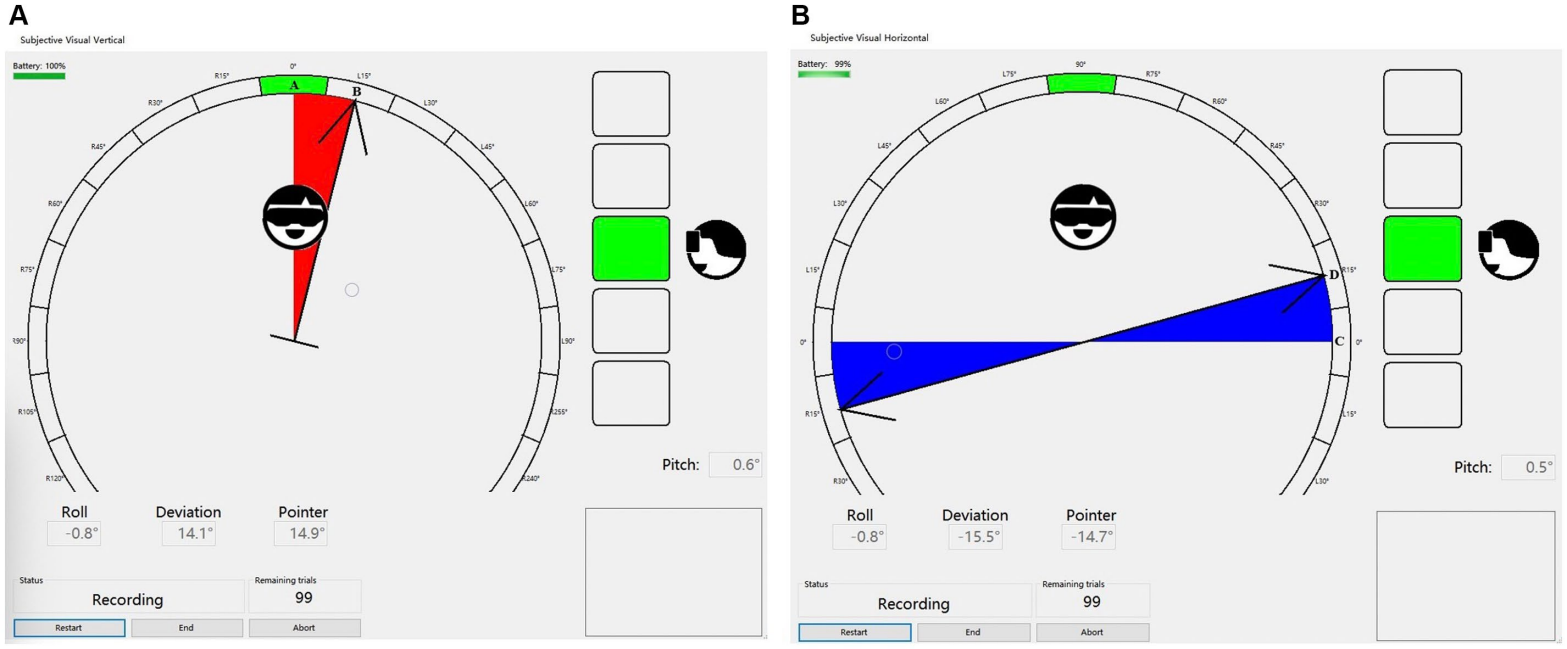
**(A)** The SVV line A is pre-set by the instrument, while the subject confirms the final SVV line B after adjusting the rotating rod. The angle between these two lines represents the degree of SVV deviation. **(B)** The SVH line C is pre-set by the instrument, while Line D denotes the SVH line confirmed by the subject after adjusting the rotating rod. The angle between these two lines is referred to as SVH deviation.

The clinical evaluation index was determined by the angle of deviation between the subject-determined SVV and SVH lines and the instrument-preset SVV and SVH lines (0°) (Figures 1A,B).

### Statistical analysis

The statistical analysis of SVV and SVH data for each group was conducted using IBM SPSS Statistics, version 25 (IBM Corporation, Armonk, NY, United States). Firstly, descriptive statistics were conducted for each group of data, followed by tests for normality and homogeneity of variance. Data that met the criteria of normality and homogeneity were analyzed using one-way ANOVA, while Kruskal-Wallis H test was employed for non-conforming data. In the event of significant differences, group comparisons are conducted and adjusted using Holm-Bonferroni multiple comparisons. The statistical significance level adopted in this study was set at α = 0.05.

## Results

The data for the acoustic neuroma group are presented for each patient (Tables 1, 2). The healthy adults’ data are presented for each subject, and mean and SD (Tables 3, 4).

**Table 1:** Subjective visual vertical (SVV) at varying degrees of static head tilt in patients with acoustic neuroma.

Parameters	R45	R30	R15	0	L15	L30	L45	PdP	Koos	Side of the tumor
Patient 1	−9.9	−7.2	−2.2	−1.4	−1.1	−0.8	2.2	−0.9	II	L
Patient 2	2.1	1	2	0.6	1	0.9	−4.2	3	IV	R
Patient 3	−25	−17.8	−9.9	−1.6	5.6	14.9	19.2	−3.7	II	R
Patient 4	−30.3	−33.7	−9.4	−0.4	2.1	11.7	16.6	1.4	II	L
Patient 5	−14.8	−16.6	−6.6	−9.2	0.5	10.3	4.7	−0.7	IV	R
Patient 6	−9	−11.8	−5.5	−2	−1.6	1.2	5.7	−7.6	III	L
Patient 7	−18.7	−16.3	−9.9	−5.3	−2.3	3.8	6.6	−4.5	III	L
Patient 8	0.2	−5.8	−6.4	−1.5	−4.1	−9.3	−7.5	−0.9	II	L
Patient 9	15.2	12.1	−3	−6.6	−11.3	−13.9	−6.4	−5.2	IV	L
Patient 10	0.1	12.8	8.3	2.7	3.6	16.4	24	3.9	II	R
Patient 10	−15.9	−5.6	−2.3	1.7	2.7	8.1	4.7	1.2	III	R
Patient 12	11.4	5.1	4.6	−1.2	−2.7	−6.4	−21.5	−2.5	II	R
Patient 13	−7.7	−10.2	−5.4	−5.6	−6.2	−1.5	6.7	−4.5	IV	R
Patient 14	−19.3	−19.8	−13.6	−6.4	−2.9	8.4	2.7	−6	I	R
Patient 15	3.8	5.5	3.3	0.8	−0.1	−3	3.2	0.8	III	R
Patient 16	−8.2	−5.4	−2	−1.5	−2.3	2.7	−0.4	−0.8	II	R
Patient 17	−6.2	−0.3	−0.6	−2.4	−3.4	3.5	18.3	−3	II	L
Patient 18	−24.3	−25.7	−10	−6.7	−4.6	0.5	13.8	−7.8	III	R
Patient 19	−2.2	8.6	2.1	−1.1	−4.1	−5.1	18	0.2	III	L
Patient 20	−19.9	−18.5	−10.4	−8	−16.3	−2	12.3	−7.6	III	L
Patient 21	−11.6	−3.1	−2.2	−1.9	−1.5	8.2	10	−6.4	II	R
Patient 22	−2.6	−3.5	0.6	−0.1	−0.6	−0.7	−6.8	0.5	IV	R

PdP, Proprioception deprivation; R, Right; L, Left; −, left; +, right.

**Table 2:** Subjective visual horizontal (SVH) at varying degrees of static head tilt in patients with acoustic neuroma.

Parameters	R45	R30	R15	0	L15	L30	L45	PdP	Koos	Side of the tumor
Patient 1	−3.1	−11.2	−8.6	−2.3	−2.1	−4.6	−6.5	−2.2	II	L
Patient 2	3.4	6.7	1	2.5	0.1	−5.3	−5.7	3.5	IV	R
Patient 3	−32.7	−17.6	−11.5	0.4	7.7	19.2	21.6	1.8	II	R
Patient 4	−7.4	−1.8	0.1	−1.4	−0.4	2.5	15.6	0.2	II	L
Patient 5	−37.3	−29.9	−9.1	−3.5	−2.3	3.2	6.4	0.3	IV	R
Patient 6	−2.2	−4.6	−8.4	−9.5	−4.8	−5.7	14.5	−6.7	III	L
Patient 7	−17.8	−2.4	−6.5	−3.1	0.8	5.2	2.7	−0.2	III	L
Patient 8	−7.6	−15.1	−4.3	−2	−1.3	−0.1	2.3	−2	II	L
Patient 9	−3.9	−0.2	−6.9	−4.7	−15.9	−7.7	−9.6	−3.5	IV	L
Patient 10	−9.6	−1.1	3.2	6.3	2	5.1	9.4	1.6	II	R
Patient 10	−9.1	−4.9	−4.4	2.9	1.1	5.1	2.4	2.4	III	R
Patient 12	11.2	8.7	8.7	−1.4	3.5	2.5	−12.7	8	II	R
Patient 13	−8.4	−3.5	−1.2	−5.9	0.2	2.3	11.5	−4.9	IV	R
Patient 14	−20.4	−13.6	−3.1	−4.2	−6.3	7.3	8.2	−3.3	I	R
Patient 15	−0.4	−1.5	3.7	0.1	5.1	−5.2	8.1	1.2	III	R
Patient 16	−9.1	−8	−7.8	−0.7	−0.8	3.4	5.7	−1.5	II	R
Patient 17	−3.6	6.4	7.4	2.4	−4.1	−5.7	1	−3	II	L
Patient 18	−39.4	−31.9	−17.3	−6.7	−4	0.4	9.5	−6.7	III	R
Patient 19	27.3	23	11.6	1.1	3	−9.4	7.2	0.3	III	L
Patient 20	−46.1	−30.6	−12.5	−0.4	13.4	27.8	48	−3.4	III	L
Patient 21	−4.3	−3.2	−5.4	−3.6	0.4	−1.3	−3.6	−5.1	II	R
Patient 22	−8.4	−1.7	−2.9	−3.2	−5.5	−7.9	1.1	−3.4	IV	R

PdP, Proprioception deprivation; R, Right; L, Left; −, left; +, right.

**Table 3:** Subjective visual vertical (SVV) at varying degrees of static head tilt in healthy adults.

Parameters	R45	R30	R15	0	L15	L30	L45	PdP
1	−2.5	0.9	−3.6	−0.5	−2.2	−7.3	0.9	−1.1
2	15.5	11.2	6.4	−1.6	−4.6	−2.5	10.1	−0.5
3	1.5	0.5	0.9	1.9	8.9	7.8	10.6	1.2
4	−3.1	−9	−1.4	−1.2	1.1	3.1	5.2	−1.7
5	−1.6	−1.8	−2.6	−1	1.4	2.7	7.5	−1.6
6	−1.4	−1.6	−0.3	−1.4	0.8	4.5	6.2	−0.6
7	0	5	0.2	1.7	2.4	9.1	23.2	1.1
8	−7.1	−4.2	−2.5	−0.2	−1.9	−1.4	−3	−2
9	−2	−7.5	−4.3	0.8	5.2	7.7	15	−0.1
10	−3.7	1.1	−2.2	−2.2	−4.1	−4.4	2.8	−3.6
11	2.7	−6	−3.7	−1.2	1.4	3.2	10.6	−1.6
12	21.2	−11.1	−6	−2.3	1	9.4	10.8	−3.1
13	−17.3	−14.7	−13.6	−2.5	1.6	5.8	4.2	−2.8
14	−6.9	−8	−5.4	−3	−3.1	−0.5	7.1	−4
15	−13.4	−13.5	−4.4	−2.5	−0.7	1.6	3.5	−1
16	−10.5	−4.1	−2.5	−0.9	−3.7	−3.1	4.6	2.6
17	3	0.9	−2.3	−1	1.6	−0.1	0.9	−1.5
18	−9.3	−6.9	−3.2	−1.6	0.2	1.4	5.4	0.8
19	−6.1	−2	−0.7	−2.3	−2.3	−2.1	1.8	−2
20	−0.3	3.5	1.4	1.1	−1.8	0.2	6.6	−3
21	−7.9	−7.6	−6	−2.2	−5.6	2.5	5.6	−3.9
22	−0.4	−1.8	−2.7	−1.4	5.4	−1.7	−6.5	−1.6
23	1.9	0.2	−1.2	−0.5	1.4	−0.5	−0.2	−0.2
24	2.4	0.3	−2	−1.9	−7.2	−4.8	0.7	−0.9
25	−2.3	3.2	1.4	−0.3	0.2	−4.1	4.7	−2
healthy adults [mean ± SD (°)]	−11.34 ± 6.97	−4.09 ± 4.33	−1.32 ± 3.46	0.04 ± 0.94	0.27 ± 3.59	2.23 ± 3.50	9.40 ± 7.24	0.09 ± 1.61

PdP, Proprioception deprivation; R, Right; L, Left; −, left; +, right.

**Table 4:** Subjective visual horizontal (SVH) at varying degrees of static head tilt in healthy adults.

Parameters	R45	R30	R15	0	L15	L30	L45	PdP
1	−2.5	0.9	−3.6	−0.5	−2.2	−7.3	0.9	−1.1
2	15.5	11.2	6.4	−1.6	−4.6	−2.5	10.1	−0.5
3	1.5	0.5	0.9	1.9	8.9	7.8	10.6	1.2
4	−3.1	−9	−1.4	−1.2	1.1	3.1	5.2	−1.7
5	−1.6	−1.8	−2.6	−1	1.4	2.7	7.5	−1.6
6	−1.4	−1.6	−0.3	−1.4	0.8	4.5	6.2	−0.6
7	0	5	0.2	1.7	2.4	9.1	23.2	1.1
8	−7.1	−4.2	−2.5	−0.2	−1.9	−1.4	−3	−2
9	−2	−7.5	−4.3	0.8	5.2	7.7	15	−0.1
10	−3.7	1.1	−2.2	−2.2	−4.1	−4.4	2.8	−3.6
11	2.7	−6	−3.7	−1.2	1.4	3.2	10.6	−1.6
12	21.2	−11.1	−6	−2.3	1	9.4	10.8	−3.1
13	−17.3	−14.7	−13.6	−2.5	1.6	5.8	4.2	−2.8
14	−6.9	−8	−5.4	−3	−3.1	−0.5	7.1	−4
15	−13.4	−13.5	−4.4	−2.5	−0.7	1.6	3.5	−1
16	−10.5	−4.1	−2.5	−0.9	−3.7	−3.1	4.6	2.6
17	3	0.9	−2.3	−1	1.6	−0.1	0.9	−1.5
18	−9.3	−6.9	−3.2	−1.6	0.2	1.4	5.4	0.8
19	−6.1	−2	−0.7	−2.3	−2.3	−2.1	1.8	−2
20	−0.3	3.5	1.4	1.1	−1.8	0.2	6.6	−3
21	−7.9	−7.6	−6	−2.2	−5.6	2.5	5.6	−3.9
22	−0.4	−1.8	−2.7	−1.4	5.4	−1.7	−6.5	−1.6
23	1.9	0.2	−1.2	−0.5	1.4	−0.5	−0.2	−0.2
24	2.4	0.3	−2	−1.9	−7.2	−4.8	0.7	−0.9
25	−2.3	3.2	1.4	−0.3	0.2	−4.1	4.7	−2
healthy adults [Mean ± SD (°)]	−1.90 ± 7.86	−2.92 ± 5.91	−2.41 ± 3.50	−1.04 ± 1.29	−0.18 ± 3.56	1.06 ± 4.44	5.53 ± 5.85	−1.32 ± 1.62

PdP, Proprioception deprivation; R, Right; L, Left; −, left; +, right.

**Figure 2:**
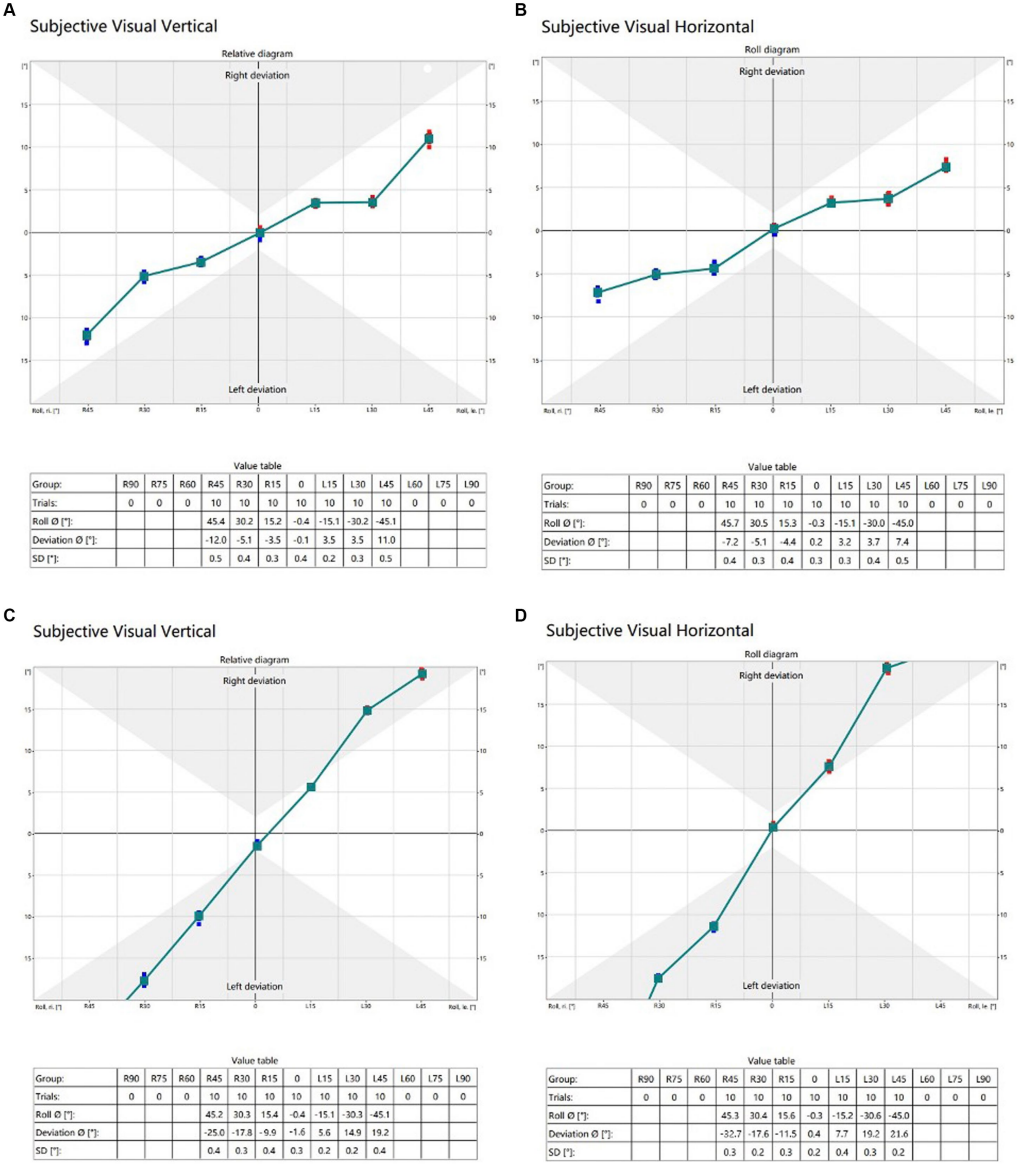
**(A)** (SVV) and **(B)** (SVH) are typical raw data in a 21-year-old healthy adult. The raw data **(C)** (SVV) and **(D)** (SVH) are representative of a 44-year-old male patient with acoustic neuroma on the right side, classified as Koos grade II. Trials refer to the number of instances where subjects were in the same head position; Roll indicates the actual tilt angle of the subject’s head position; Deviation means SVV or SVH deviation; SD denotes the standard deviation of the tilt angle of the subject’s head.

Shown in Figure 2 are typical raw data test results from a normal subject and an AN subject with the head upright and varying degrees of head tilt.

**Figure 3:**
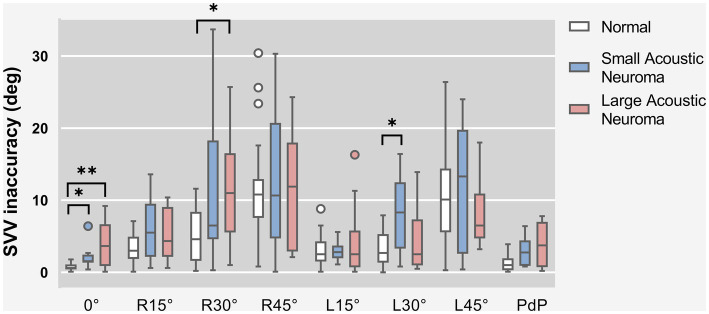
SVV statistical results of patients with unilateral acoustic neuroma (LAN group and SAN group) and healthy adults reference controls were compared under different degrees of head tilt. * and **Indicates statistically significant difference. *p < 0.05. **p < 0.01.

SVV statistical results of patients with unilateral acoustic neuroma (LAN group and SAN group) and healthy adults reference controls were compared under different degrees of head tilt (Figure 3).

Due to the failure of meeting homogeneity of variance assumptions in each group, Kruskal-Wallis H test was utilized for data comparison. The results indicated that there were statistically significant differences among the Head centered (0° tilt), Head tilt 30° to the right, Head tilt 30° to the left and PdP groups (0°: *H* = 15.098, *p* = 0.001; R30°: *H* = 8.625, *p* = 0.013; L30°: *H* = 7.483, *p* = 0.024; PdP: *H* = 7.647, *p* = 0.022). However, no significant difference was found in other head position groups. Subsequently, a pairwise comparison was conducted between the Head centered (0° tilt), Head tilt 30° to the right, Head tilt 30° to the left, and PdP groups.

Head centered (0° tilt), both the LAN and SAN groups exhibited a significantly higher deviation angle of the SVV compared to the normal group (*Z* = 3.374, *p* = 0.002 and *Z* = 2.863, *p* = 0.013, Holm-Bonferroni correction). There was no significant difference between the LAN and SAN groups (*Z* = 0.266, *p* = 1, Holm-Bonferroni correction).

Head tilt 30° to the right. The LAN group exhibited a significantly increased deviation angle of SVV compared to the normal group (*Z* = 2.731, *p* = 0.019, Holm-Bonferroni correction). No significant difference was observed between the SAN group and the normal group (*Z* = 1.872, *p* = 0.184, Holm-Bonferroni correction), while there was no statistical difference between the SAN and LAN groups (*Z* = 0.605, *p* = 1, Holm-Bonferroni correction).

Head tilt 30° to the left. Compared to the normal group, the SAN group demonstrated a significantly increased deviation angle of SVV (*Z* = 2.652, *p* = 0.024, Holm-Bonferroni correction), indicating a statistically significant difference. No significant difference was observed between the LAN group and the normal group (*Z* = 0.168, *p* = 1, Holm-Bonferroni correction). Additionally, there was no statistical difference found between the LAN and SAN groups (*Z* = 2.180, *p* = 0.088, Holm-Bonferroni correction).

After applying the Holm-Bonferroni correction to the PdP position data, it was determined that there were no significant differences in deviation angle among any of the groups (*Z* = 2.264, *p* = 0.071; SAN group - normal group: *Z* = 2.202, *p* = 0.083; SAN group-LAN group: *Z* = −0.067, *p* = 1).

**Figure 4:**
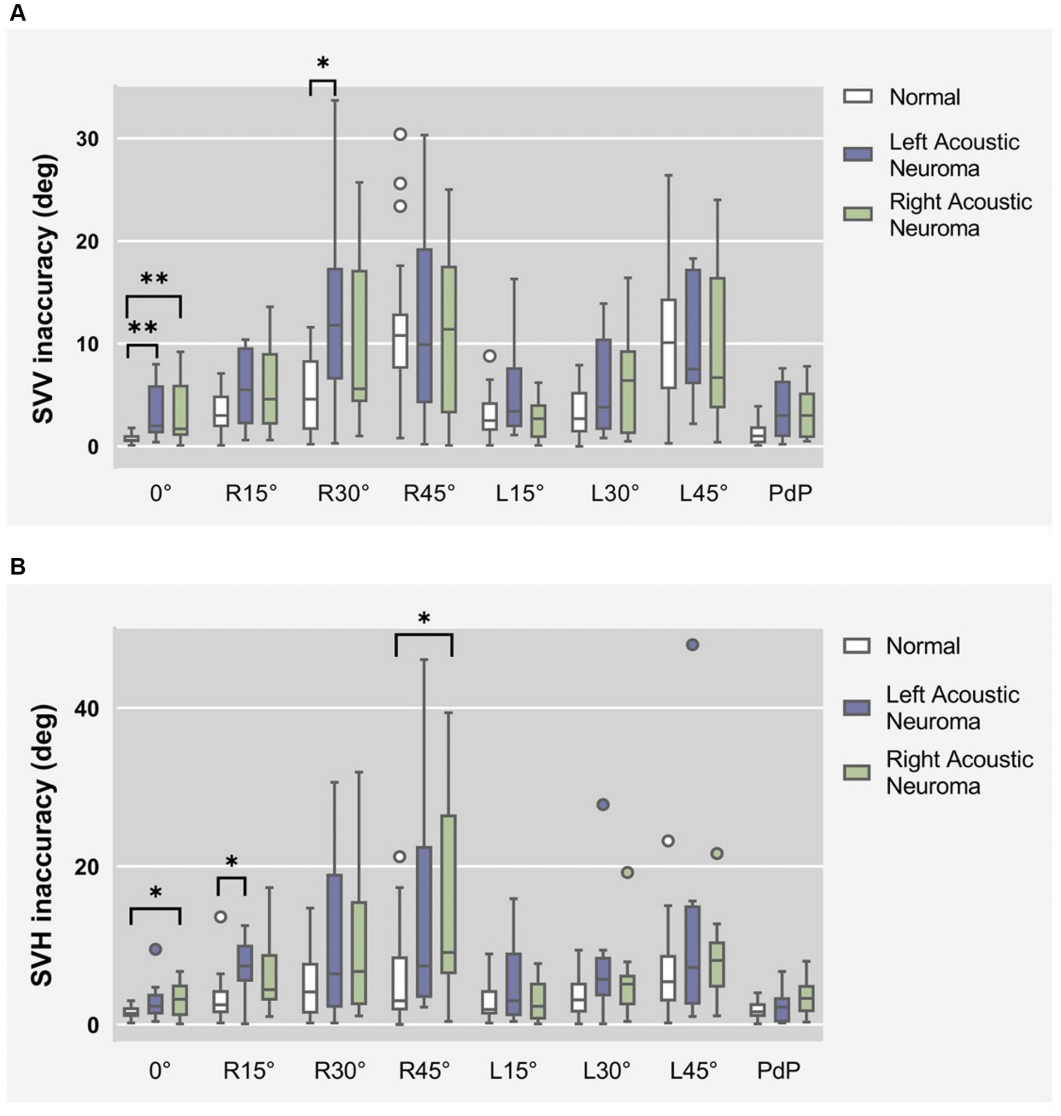
**(A)** SVV statistical results of patients with different side of acoustic neuroma (left group and right group) and healthy adults reference controls were compared under different degrees of head tilt. **(B)** SVH statistical results of patients with different side of acoustic neuroma (left group and right group) and healthy adults reference controls were compared under different degrees of head tilt. * and **Indicates statistically significant difference. *p < 0.05. **p < 0.01.

SVV statistical results of patients with different side of acoustic neuroma (Left group and Right group) and healthy adults reference controls were compared under different degrees of head tilt (Figure 4A).

Due to the failure of meeting homogeneity of variance assumptions in each group, Kruskal-Wallis H test was utilized for data comparison.

Head centered (0°tilt), compared to the normal group, the SVV deviated angle significantly increased in both the left and right groups (*p* = 0.006 and *p* = 0.005). There was no statistically significant difference between the left and right groups (*p* > 0.05).

Head tilt 30° to the right, the SVV deviated angle in the Left group exhibited a statistically significant increase compared to the normal group (*p* = 0.023).

There was no statistically significant difference in the remaining head tilt position among the groups (*p* > 0.05).

**Figure 5:**
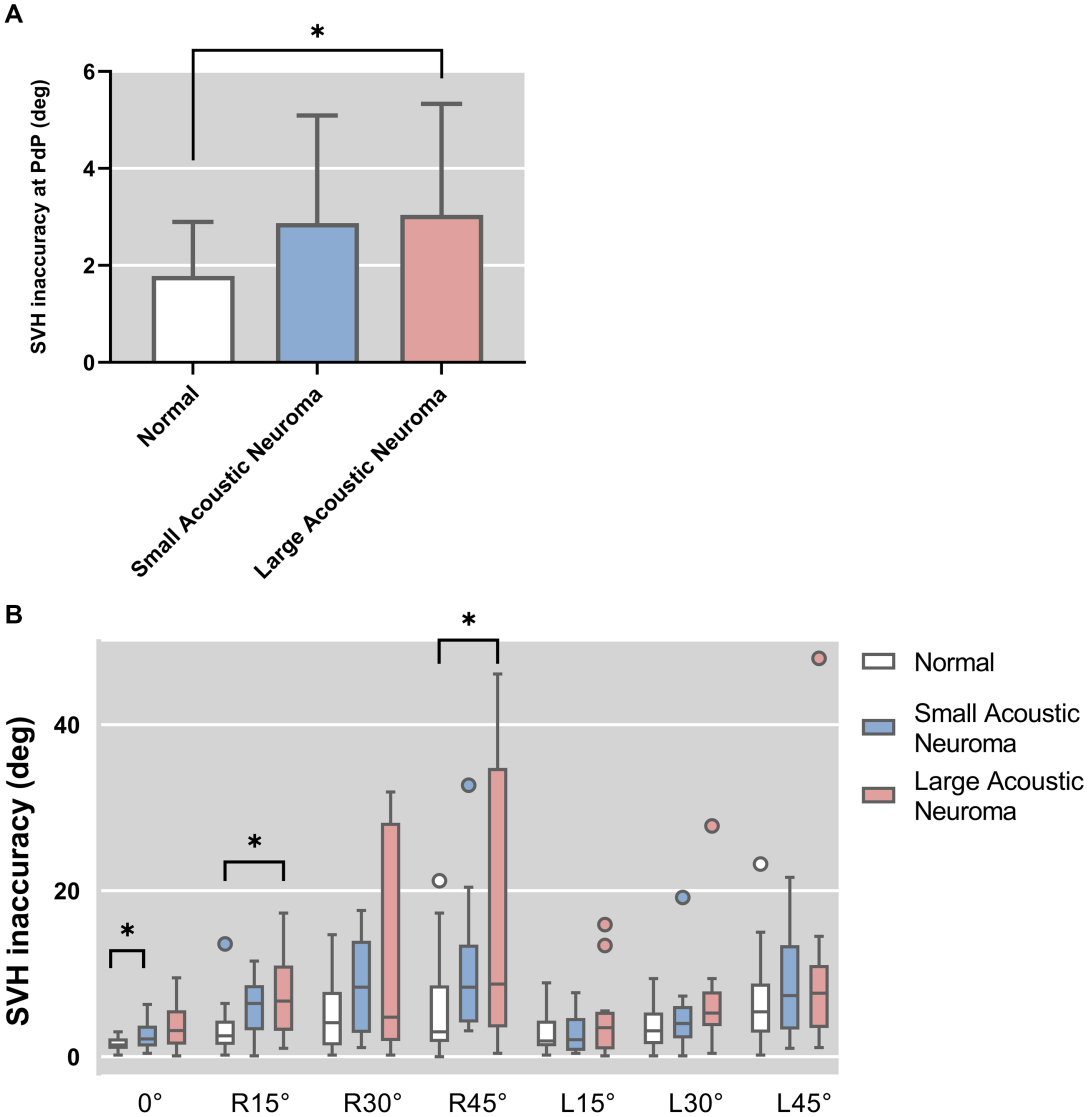
**(A)** SVH statistical results of patients with unilateral acoustic neuroma (LAN group and SAN group) and healthy adults reference controls were compared under PDP position. **(B)** SVH statistical results of patients with unilateral acoustic neuroma (LAN group and SAN group) and healthy adults reference controls were compared under different degrees of head tilt. *Indicates statistically significant difference. *p < 0.05.

SVH statistical results of patients with unilateral acoustic neuroma and healthy adults’ reference controls were compared under different degrees of head tilt (Figure 5).

Following hypothesis testing, it was determined that the PdP position data exhibited normality and homogeneity of variance, thus warranting the use of one-way ANOVA for analysis. Conversely, the remaining data did not meet these assumptions and were therefore subjected arisen using Kruskal-Wallis H test.

The results of one-way ANOVA revealed a significant difference in the deviated angle among the three groups (LAN, SAN, normal) at PdP position (*F* = 4.896, *p* = 0.012). Subsequently, pairwise comparisons were conducted.

The deviation angle of the LAN group exhibited a significant increase compared to that of the normal group (*p* = 0.010, Holm-Bonferroni correction). No statistically significant difference was observed between the SAN and normal groups (*p* = 1, Holm-Bonferroni correction), nor between the LAN and SAN groups (*p* = 0.167, Holm-Bonferroni correction).

The results of the Kruskal-Wallis H test indicated a statistically significant difference among the groups with head centered (0° tilt), 15° head tilt, and 45° right head tilt (0°: *H* = 7.814, *p* = 0.020; R15°: *H* = 9.665, *p* = 0.008; R45°: *H* = 8.393, *p* = 0.015). There was no significant difference observed in the other positions of the head. Further group comparisons were conducted between the Head-centered (0° tilt) condition and conditions with a 15° or 45° head tilt to the right.

Head centered (0° tilt). The SVH deviated angle was found to be significantly higher in the SAN group compared to the normal group (*Z* = 2.642, *p* = 0.025, Holm-Bonferroni corrected), indicating a statistically significant difference between the two groups. No significant difference was observed between the LAN group and the normal group (*Z* = 2.641, *p* = 0.281, Holm-Bonferroni corrected). Additionally, no significant difference was noted between the LAN and SAN groups (*Z* = 0.933, *p* = 1, Holm-Bonferroni corrected).

Head tilt was 15° to the right. Compared to the normal group, the LAN group exhibited a significant increase in SVH deviated angle (*Z* = 2.824, *p* = 0.014, Holm-Bonferroni corrected), indicating a statistically significant difference. No statistical difference was observed between the SAN and normal groups (*Z* = 2.098, *p* = 0.108, Holm-Bonferroni corrected), and there was no statistical difference between the LAN and SAN groups (*Z* = 0.483, *p* = 1, Holm-Bonferroni corrected).

Head tilt 45° to the right. Compared to the normal group, the LAN group exhibited a significant increase in SVH deviated angle (*Z* = 2.689, *p* = 0.022, Holm-Bonferroni corrected), while there was no statistical difference between the SAN and normal groups (*Z* = 1.846, *p* = 0.195, Holm-Bonferroni corrected) or between the LAN and SAN groups (*Z* = 0.592, *p* = 1, Holm-Bonferroni corrected).

SVH statistical results of patients with different side of acoustic neuroma (Left group and Right group) and healthy adults reference controls were compared under different degrees of head tilt (Figure 4B).

Kruskal-wallis H test results show that Head centered (0° tilt), the SVH angle between the Right group and the normal group exhibited a statistically significant increase (*p* = 0.039).

Head tilt 15° to the right, the SVH deviated angle in the Left group exhibited a significantly higher value compared to that in the normal group (*p* = 0.011), indicating a statistically significant difference.

Head tilt 45° to the right, the SVH deviated angle in the Right group exhibited a statistically significant increase compared to the normal group (*p* = 0.017).

There was no statistically significant difference in the remaining head tilt position among the groups (*p* > 0.05).

## Discussion

The primary aim of our study was to ascertain whether there existed any disparities in the absolute deviated values of subjective visual vertical and horizontal between individuals afflicted with acoustic neuroma and healthy young adults, across eight distinct degrees of head tilt (Head centered (0° tilt), PdP, Head tilt 15°, 30°, 45° to the left and Head tilt 15°, 30°, 45° to the right). The findings indicate that there were significant statistical differences in the absolute deviated values of subjective visual vertical (SVV) and subjective visual horizontal (SVH) between patients with acoustic neuroma and healthy young adults at different head tilt angles in part. These results also suggest that the choice of SVV or SVH tests as pathological indicators of vestibular function may differ.

The mechanism underlying static subjective visual vertical and horizontal perception is predicated on the degree of disparity between information received by the bilateral vestibular system and that processed by the central nervous system. Based on literature reports, previous studies have demonstrated that the range of SVV deviation in the normal population is typically within ±2.5° when individuals are seated upright (Akin et al., 2011; Dakin et al., 2018). However, Michelson et al. utilized the virtual reality system to assess subjective visual vertical (SVV) in 15 healthy adults and observed a deviation of 3.8° when the head was in an upright position at 0°, while SVV deviated by 14.67° and 11.86° respectively when the head was tilted to the right or left at an angle of 45° (Michelson et al., 2018). Cheng et al. (2022) utilized VR technology to assess SVV and SVH values in 80 healthy adults, with a head tilt of 0° resulting in SVV values of −0.13° ± 1.61 and SVH values of −0.94° ± 1.81. This may be attributed to variations in laboratory conditions, equipment, operators, and other factors. Each laboratory should establish its own standard reference range of values to develop a standardized protocol for the efficient and expedient assessment of utricle function. Therefore, in this study, we recruited 25 healthy young adults to investigate eight static head-tilt positions (Head centered (0° tilt); PdP; leftward head tilts of 15°, 30°, and 45°; rightward head tilts of 15°, 30°, and 45°) with the aim of establishing reference ranges for SVV and SVH in our laboratory. The mean age of the healthy young individuals selected as reference values is 19 years old, which differs from the mean age of 42 years old in the AN patient group. However, Sun et al. (2014) reported an optimal diagnostic threshold of 2° when testing SVV in a group with an average age of 77 years, leading them to conclude that SVV is not dependent on age. Given the lower prevalence of underlying conditions affecting vestibular function among healthy young adults, it is reasonable to utilize this demographic as a reference group.

SVV and SVH serve as valuable indices for assessing spatial disorientation in patients with peripheral and/or central vestibular injuries (Ashish et al., 2017). The subjective visual vertical (SVV) evaluates the function of the utricle, and the determined deviation angle of SVV provides a measure of utricular function. Previous research has indicated that the ocular torsional response is linked to otolithic disorders and, as a result, abnormal SVV tilt (Funabashi et al., 2015). Therefore, a tilt of the SVV toward the more severely affected ear may be associated with an ipsilateral ocular torsional reaction, which could be caused by decreased neuronal activity in the vestibular nucleus on that side due to reduced otolithic input (Bronstein et al., 2003). Wang et al. (2021) demonstrated excellent test–retest reliability of the subjective visual vertical deviation during lateral head tilts as measured with the virtual reality system, providing support for further investigation into the diagnostic utility of head-tilt SVV in various vestibular disorders. Cnyrim et al. (2007) reported that both peripheral and central lesions can impair the accuracy of subjective visual vertical (SVV) when subjects’ heads are in an upright position. Our findings support this previous research. In the Head centered (0° tilt) position, the AN patient group exhibited significantly higher absolute deviated values of SVV and SVH compared to healthy young adults. This suggests that when expressing absolute values of SVV and SVH deviation, the accuracy of judgments for both parameters is decreased in AN patient. Additionally, our findings indicate that the absolute mean deviation values of SVV and SVH exhibit an upward trend as head tilt increases within the range of 0–45°. When the head is tilted at a 30° angle, individuals with AN exhibit significantly higher deviations in their subjective visual vertical (SVV) values compared to healthy controls. The head was tilted 15° and 45° to the right, there is a significant increase in SVH absolute deviation values observed in the AN group relative to healthy young adults. These findings suggest that static head tilts may exacerbate perceptual asymmetries resulting from peripheral or central vestibular pathway dysfunction. There could be two possible explanations for this phenomenon. One factor is the increase in sensory noise from vestibular or somatosensory input to otolith organs during lateral head tilt. Another factor is that the subject’s head tilt causes an increase in proprioceptive input weight of neck muscle tissue, resulting in interference with multisensory integration (De Vrijer et al., 2009; Tarnutzer et al., 2009; Clemens et al., 2011). Patients with AN may have peripheral and/or central vestibular damage and have disturbances of multi-sensory integration, which may lead to increased SVV and SVH deviated value.

In this study, we also wanted to investigate the impact of acoustic neuroma size on SVV and SVH absolute deviation values in patients with varying degrees of head tilt. The findings indicated that there was no statistically significant disparity in the SVV and SVH absolute deviation values between the LAN and SAN groups among acoustic neuroma patients at all eight head tilt positions. These findings suggest that tumor size has little impact on the absolute deviation values of SVV and SVH, as well as the gravity sensing function in patients. Our hypothesis posits that the observed phenomenon may be attributed to central vestibular compensation mechanisms and pre-existing differences in vestibular function prior to surgery. Compensation is a vestibular system function that parallels the central nervous system. Ashish et al. (2017) conducted a study on patients with vestibular migraine and observed no significant difference in static subjective visual vertical (SVV) values compared to controls, indicating that these patients may have achieved tonic vestibular compensation during symptom-free periods. Due to the slow growth of acoustic neuromas, it is believed that central vestibular compensation may occur during their development. Moreover, among our group of patients diagnosed with acoustic neuroma, 10 (45.45%) exhibited symptoms of dizziness, vertigo, or impaired balance which resulted in variations in vestibular function across individuals and consequently confounded the impact of tumor size on absolute deviation values for both SVV and SVH measurements.

Furthermore, a significant difference in SVH deviation was observed between the LAN group and the healthy youth group when subjects’ heads were tilted 15° and 45° to the right, whereas no statistically significant difference was found between the two groups when subjects’ heads were tilted 15° and 45° to the left. When analyzing the effect of acoustic neuroma side on SVV and SVH absolute deviation values at various degrees of head tilt, we observed that at 0°, the SVV absolute deviation values exhibited statistically significant differences compared to those in the normal group, regardless of whether the acoustic neuroma was situated on the left or right side. However, only in the tumor group with right-sided localization did we observe a statistically significant difference in SVH deviation compared to the normal group. When the head was tilted to different degrees, it was found that the absolute deviation values of SVV and SVH were statistically significant only when the head was tilted to the right side, which was similar to the analysis results of tumor size. Among the 22 patients with acoustic neuroma included in our study, 9 were diagnosed on the left side, including 5 in the LAN group, with 1 case (20%) classified as grade IV. On the right side, there were a total of 13 cases, out of which 7 were part of the LAN group, including 4 cases (57%) categorized as grade IV. It can be seen that the proportion of patients with large tumors in the right-side group is relatively high. Therefore, we considered that the influence of tumor side on SVV and SVH deviation may be somewhat related to tumor size. The Left group and the Right group did not exhibit any statistically significant differences across all head positions. Ferreira et al. (2017) has reported that in cases of unilateral extrajudicial disease or neuro-nucleus damage, SVV is tilted toward the affected side. However, when the injury site is located in or above the unilateral pontine, SVV tilts to the healthy side. In instances where there is thalamic or cerebellar dental nucleus injury present, SVV may tilt in either direction of the affected or healthy side. The mechanism of SVV deviation remains unclear when the range of lesions caused by acoustic neuroma involves damage to the thalamic or cerebellar dental nucleus. Therefore, it is imperative to further validate this finding through future studies with a larger study population in order to compare the different sides of acoustic neuroma.

In this study, we assessed the SVV and SVH absolute deviation values of both AN patients and healthy young adults at varying degrees of head tilt. Additionally, we examined the impact of proprioception by testing the SVV and SVH values with all subjects standing on a foam cushion in a neutral head position (Proprioception deprivation, PdP, 0° tilt). The results indicate that only the LAN group exhibited a statistically significant increase in SVH deviation Angle compared to the healthy young people group, while no significant difference was observed between the remaining SVH groups and the SVV groups. These findings suggest that proprioception plays a crucial role in determining SVH outcomes when acoustic neuroma exceeds 2 cm in size, whereas it has little impact on SVV and SVH deviation value when acoustic neuroma is smaller than 2 cm.

In conclusion, we believe that acoustic neuroma exerts varying degrees of influence on patients’ gravity sensing function. However, there appears to be little correlation between the degree of gravity sensitivity impairment and tumor size. This suggests that we should pay attention to the postoperative follow-up of patients with acoustic neuroma and the evaluation of vestibular rehabilitation effect in clinical work. The central compensation of AN patients with unilateral vestibular dysfunction after surgery can be promoted and improved through specific vestibular rehabilitation program (Perez et al., 2006). The study by Hrubá1et al. showed that the compensatory effect of short-term vestibular rehabilitation on vestibular function after surgery for vestibular schwannoma can be evaluated by SVV deviation (Hrubá et al., 2019). Therefore, it is possible to follow up patients after surgery and test SVV and SVH, gradually personalized adjusting the patient’s vestibular rehabilitation plan strategy as the deviation values of SVV and SVH improvement. Meanwhile, for patients opting for conservative treatment, it is imperative to monitor the dynamic changes in vestibular function, assess alterations in gravity sensing and auditory nerve function, and seize timely opportunities for surgical intervention.

## Limitation

Although our study yielded encouraging results, it is important to note its limitations. Such as we did not collect postoperative SVV and SVH results of AN patient which prevented us from confirming whether there was a reversible recovery of impaired gravity sensing function. In addition, the small sample size of acoustic neuroma patients in our study may affect the efficacy of statistical testing. In the future, we will continue to expand and enrich the sample for related research.

## Data availability statement

The original contributions presented in the study are included in the article/supplementary material, further inquiries can be directed to the corresponding authors.

## Ethics statement

The study was approved by the ethical review board of Sun Yat-sen Memorial Hospital of Sun Yat-sen University. Written informed consent was waived because of this retrospective analysis.

## Author contributions

LZ: Writing – original draft, Data curation, Formal analysis. SO: Methodology, Writing – original draft. LC: Data curation, Formal analysis, Writing – original draft. HH: Conceptualization, Data curation, Formal analysis, Writing – original draft. YO: Conceptualization, Supervision, Writing – review & editing. XT: Conceptualization, Writing – review & editing.
